# Are ADC values of readout-segmented echo-planar diffusion-weighted imaging (RESOLVE) correlated with pathological prognostic factors in rectal adenocarcinoma?

**DOI:** 10.1186/s12957-018-1445-z

**Published:** 2018-07-12

**Authors:** Cui Tang, Mou-bin Lin, Jin-lei Xu, Lan-hua Zhang, Xiao-ming Zuo, Zhong-shuai Zhang, Meng-xiao Liu, Jin-ming Xu

**Affiliations:** 1 Department of Radiology, Tongji University Affiliated Yangpu Hospital, No. 450 Tengyue Road, Shanghai, 200090 China; 2 Department of General Surgery, Tongji University Affiliated Yangpu Hospital, Shanghai, 200090 China; 3 Department of Pathology, Tongji University Affiliated Yangpu Hospital, Shanghai, 200090 China; 4 SIEMENS Healthineers Ltd., Shanghai, China

**Keywords:** Rectal cancer, MRI, Apparent diffusion coefficient, Pathology, Prognosis

## Abstract

**Background:**

Diffusion-weighted imaging (DWI) and apparent diffusion coefficient (ADC) values as imaging biomarkers of rectal cancer are currently a hot research spot. The use of ADC values for preoperative judgment of pathological features in rectal cancer has been generally accepted. The image quality evaluation of conventional diffusion is severe deformation, and the measurement of ADC values can easily lead to bias. Readout-segmented echo-planar diffusion-weighted imaging (RESOLVE) provides high signal-to-noise ratio images and significantly reduces distortions caused by magnetosensitive effects. The purpose of this study was to explore the correlations between ADC values of RESOLVE and pathological prognostic factors in rectal adenocarcinoma.

**Methods:**

We collected pathological data of 89 patients with pathologically confirmed rectal adenocarcinoma who directly underwent surgical resection without receiving adjuvant therapy. The patients were grouped according to the pathologic type, gross classification, degree of differentiation, TN stage, and immunohistochemical expression of epidermal growth factor receptor (EGFR).

**Results:**

RESOLVE ADC values of rectal cancer were measured at *b* = 800, and correlations between the RESOLVE ADC values obtained in different groups were analysed. We found that RESOLVE ADC values in the ulcer-type group were significantly higher than those in the eminence-type group.

**Conclusion:**

RESOLVE ADC values in different pathologic types of rectal cancer were significantly different. RESOLVE ADC values in the EGFR-positive group were significantly lower than those in the EGFR-negative group. There was no significant difference in RESOLVE ADC values between different degrees of pathologic differentiation, TN stages, and positive or negative lymph nodes. The quantitative description of RESOLVE ADC values could be used to assess the biological behaviour of rectal adenocarcinoma.

## Background

Colorectal cancer (CRC) is a common malignant tumour of the digestive system with the incidence increasing yearly. According to the latest data released by Cancer Statistics in China 2015, the incidence of CRC ranks fourth in women and fifth in men of all cancers in China and CRC ranks fifth in mortality in both men and women. Of all CRC cases, rectal cancer accounts for about 50% [1]. Therefore, rectal surgeons are often confronted with the complexity of patient conditions and the challenge to improve the prognosis in their clinical diagnosis and treatment. Understanding tumour pathological features before treatment has important guiding value in formulating the clinical treatment plan and predicting the prognosis. It is common knowledge that the pathological type, degree of differentiation, depth of infiltration, cancerous tissue involvement, and the presence or absence of regional lymph node (LN) metastasis can to some extent reflect the degree of tumour malignancy and predict the invasiveness and prognosis of a tumour [2]. However, direct assessment of the tumour’s biological behaviour by biopsy is invasive and time-consuming with poor reproducibility. For this reason, diffusion-weighted magnetic resonance imaging (DWI-MR) has been recognised as an indirect test with great application potential. DWI is the most ideal method for examining water molecule diffusion in the tissue in vivo. It can also be used to assess water molecule diffusion by measuring apparent diffusion coefficient (ADC) values. Additionally, epidermal growth factor receptor (EGFR), a multi-functional glycoprotein extensively distributed on human tissue cell membranes, is highly expressed in various human cancers, playing important roles in promoting the development of tumours, proliferation and migration of tumour cells, and tumour angiogenesis, and inhibiting apoptosis of tumour cells [3]. EGFR is also an important target in the treatment of metastatic rectal cancer [4]. In recent years, many studies have made efforts to utilise DWI to predict the characteristics of tumour pathological subtypes [5], cellular structures, [6] and invasiveness [7] and predict the therapeutic effect [8]. There are also studies focusing on the correlation of DWI with pathological prognostic factors of rectal cancer with respect to the tumour pathological type, degree of differentiation, and TN stage [9–11]. Conventional DWI uses single-shot echo-planar imaging technique to acquire *k*-space data. However, such sequence trajectory is very sensitive to susceptibility artefacts, so that the resulting geometric distortion contaminates the quality of the DW images [12–15]. In order to reduce the image distortion, a new acquisition method, readout-segmented echo-planar diffusion-weighted imaging (RESOLVE), was proposed, which uses multiple shots EPI along the readout direction to sample *k*-space data. There are few studies focusing on the correlation of RESOLVE ADC values with pathological prognostic factors in rectal cancer, and the results obtained are also controversial. In addition, there are even fewer studies about the correlation of RESOLVE ADC values with the expression of EGFR in rectal cancer. The purpose of this study is to explore the relationship between ADC values obtained by RESOLVE and the main pathological prognostic factors of rectal cancer and to assess whether such ADC values correlate with the positive expression of EGFR.

## Methods

### Subjects

**Fig. 1:**
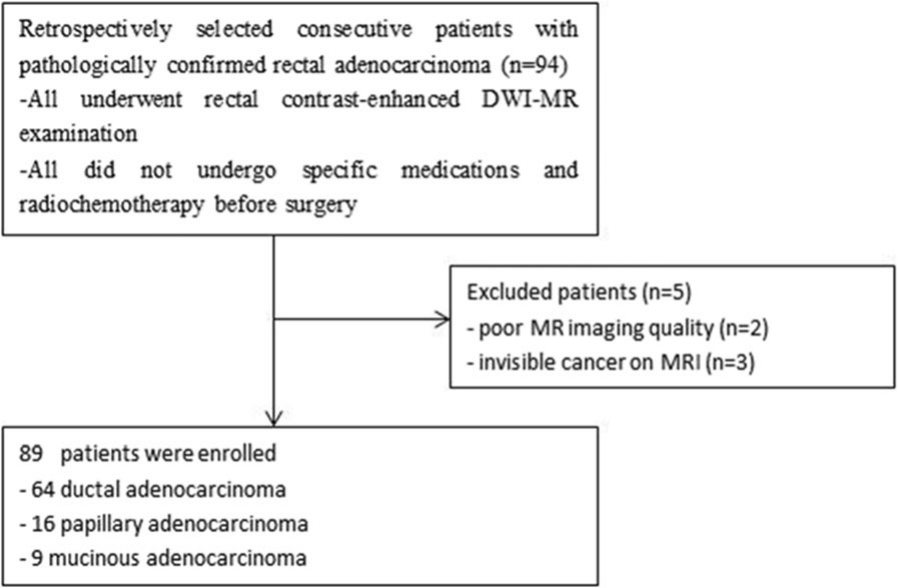
Flow diagram of patients with rectal adenocarcinoma

Pathological data of 94 patients who were confirmed as having rectal adenocarcinoma (RAC) by pathology and received surgical resection at Tongji University Affiliated Yangpu Hospital (China) between December 2014 and December 2016 were reviewed retrospectively. None of the patients had received specific medications and radiochemotherapy before surgery. All the patients underwent contrast-enhanced DWI-MR examination within a week before surgery. Of the 94 initially recruited patients, 5 were excluded, 2 because of poor MR imaging quality, and 3 had small lesions that could not be displayed clearly on MRI because they had undergone enteroscopic biopsy before MRI examination. As a result, a total number of 89 patients were included for analysis. Pathologically, there were 64 cases of ductal adenocarcinoma, 16 cases of papillary adenocarcinoma, and 9 cases of mucinous adenocarcinoma (Fig. 1).

All participants signed a written informed consent in this study.

### Research methods

#### MRI instruments and methods

Using a 3.0T MR scanner (Magnetom Skyra; Siemens Healthcare, Erlangen, Germany) with a pelvic phased-array coil, whole-pelvic scans were performed from 10 cm below the symphysis pubis to the level of the sacral promontory; the used routine MRI sequences were sagittal T2WI, transverse T2WI, and T1WI with the following scan parameters: sagittal T2WI (TR = 1000 ms, TE = 89 ms, and FOV 420 mm × 420 mm), transverse T2WI (TR = 1600 ms, TE = 96 ms, matrix = 320 × 200, and FOV = 380 mm × 270 mm), transverse T1WI (TR = 600 ms, TE = 20 ms, matrix = 360 × 270, and FOV = 320 mm × 260 mm). In addition, DWI was also performed using RESOLVE technique, diffusion-sensitised factor *b* = 0, 50, and 800 s/mm^2^ (TR = 5200 ms, TE = 67 ms, thickness = 3 mm, matrix = 112 × 140, and FOV = 230 mm × 230 mm), and T1WI contrast-enhanced scan (TR = 6.5 ms, TE = 2.2 ms, thickness = 3 mm, matrix = 156 × 320, and FOV = 260 mm × 320 mm).

#### Image post-processing and ADC value assessment

The RESOLVE information was post-processed to obtain the corresponding ADC images, which were then analysed by two experienced radiologists (with 4 years of experience in MRI diagnosis) who were blinded to the clinical status of the patients and the pathological diagnoses. The optimal layer of the tumour displayed on RESOLVE (*b* = 800 s/mm^2^) was found, and the region of interest (ROI) was placed on the main tumour body of the corresponding ADC image to obtain the ADC value of the region. According to the morphology of the tumour, the ROI was circled on the solid portion of the tumour; the ADC values of the circular or oval ROI on three consecutive levels that displayed the tumour lesion were measured by avoiding the haemorrhagic and necrotic areas. The mean of the three ADC measurements was used as the final result. It should be noted that the size of ROI should be basically the same with the maximum diameter not exceeding 5 mm and that ROI should be selected as much as possible at the central area of the solid tumour.

#### Analysis of the pathological prognostic factors

The pathological prognostic factors included the gross classification of the tumour, histological type, pathologic differentiation, TN stage, and cancer nodules. Immunohistochemistry included the expression of EGFR. According to the pathological gross classification, the included cases were classified as eminence type, ulcer type (limited ulcer type and infiltration ulcer type), and diffuse type, and pathologically as ductal adenocarcinoma, papillary adenocarcinoma, and mucinous adenocarcinoma. The eminence type refers to the main body of the tumour protruding into the lumen of the intestine. The ulcer type is endogenous growth, which refers to the infiltration of the tumour into the intestinal wall and the formation of ulcers; the infiltrating type is also endogenous growth, which means that the tumour is diffusely infiltrated into each layer of the intestinal wall, and the surface may have ulceration. According to the glandular and ductal morphological features, rectal cancer was classified as well, moderately, or poorly differentiated. TNM staging was evaluated according to the seventh edition of the American Joint Committee on Cancer (AJCC7). The tumours were staged as T1, T2, T3, and T4 depending on the depth of tumour infiltration. N staging was based on the existence or non-existence of regional lymph node (LN) metastasis, and at the same time, the presence or absence of cancer nodules was observed. The cancer nodules are defined as tumours in the subserosal, mesenteric, and peritoneal-covered colon and the surrounding tissues of the rectum. According to immunohistochemical staining, the expression of EGFR was classified as positive or negative.

### Statistical analysis

RESOLVE ADC values of the measurement data were reported as mean ± standard deviations (SD). Differences in RESOLVE ADC values relative to the tumour gross classification, histological type, pathologic differentiation, and T stage were compared by single factor analysis of variance. Interobserver variabilities of the ADC values of the two radiologists for the tumour were analysed according to the Bland-Altman method and by calculating the intra-class correlation coefficients. Differences in RESOLVE ADC values between the groups with or without regional LN metastasis, cancer nodules, and positive or negative EGFR expression were compared using the independent sample *t* test. For all statistical tests, *P* values less than 0.05 was considered statistically significant. For statistically significant prognostic factors, the threshold values of RESOLVE ADC of the corresponding tumours were obtained by using ROC curves. All statistical analyses were performed using SPSS16.0 software.

## Results

### Interobserver variability

The interobserver reproducibility was excellent for the tumour ADC values measured by the two radiologists, and the intra-class correlation coefficient was 0.86.

### Clinicopathological characteristics of RAC

Among the 89 enrolled patients in this study, 62 were men and 27 were female. The mean age was 63.9 years (range 32–86). Among the 89 RAC cases, there were 56 cases of ulcer type and 33 cases of eminence type, 64 cases of ductal adenocarcinoma, 16 cases of papillary adenocarcinoma, and 9 cases of mucinous adenocarcinoma. Regarding the degree of differentiation, the cases of high differentiation, medium differentiation, and low differentiation were 16, 63, and10 cases, respectively. Regarding T stage, there were 5, 36, 46, and 2 cases of T1, T2, T3, and T4, respectively. There was no diffuse-type rectal cancer patient in this group. As the number of T4 cases was small, we merged T3 and T4 as one T3–4 group totalling 48 cases. In terms of N staging, 33 cases had regional LN metastasis (N+), and 56 cases had no regional LN metastasis (N0). Cancer nodules were detected in 10 cases, and no cancer nodule was detected in 79 cases. Immunohistochemistry showed positive EGFR in 63 cases and negative EGFR in 26 cases (Table 1).

**Table 1:** Clinicopathological characteristics of the patients

Clinicopathological characteristics		*n* percentile (%)
Gender	Male	62 (69.7)
	Female	27 (30.3)
Age (years)	≤ 60	31 (34.8)
	> 60	58 (65.2)
Gross classification	Ulcer	56 (62.9)
	Eminence	33 (37.1)
Histological type	Tubular	64 (71.9)
	Papillary	16 (18.0)
	Mucinous	9 (10.1)
Pathologic differentiation	High	16 (18.0)
	Moderate	63 (70.8)
	Poor	10 (11.2)
T stage	T1	5 (5.6)
	T2	36 (40.5)
	T3	46 (51.7)
	T4	2 (2.2)
N stage	N0	56 (62.9)
	N+	33 (37.1)
Cancer nodule	Positive	10 (11.2)
	Negative	79 (88.8)
EGFR	Positive	63 (70.8)
	Negative	26 (29.2)

### Correlations of RESOLVE ADC values with pathological prognostic factors in RAC

**Fig. 2:**
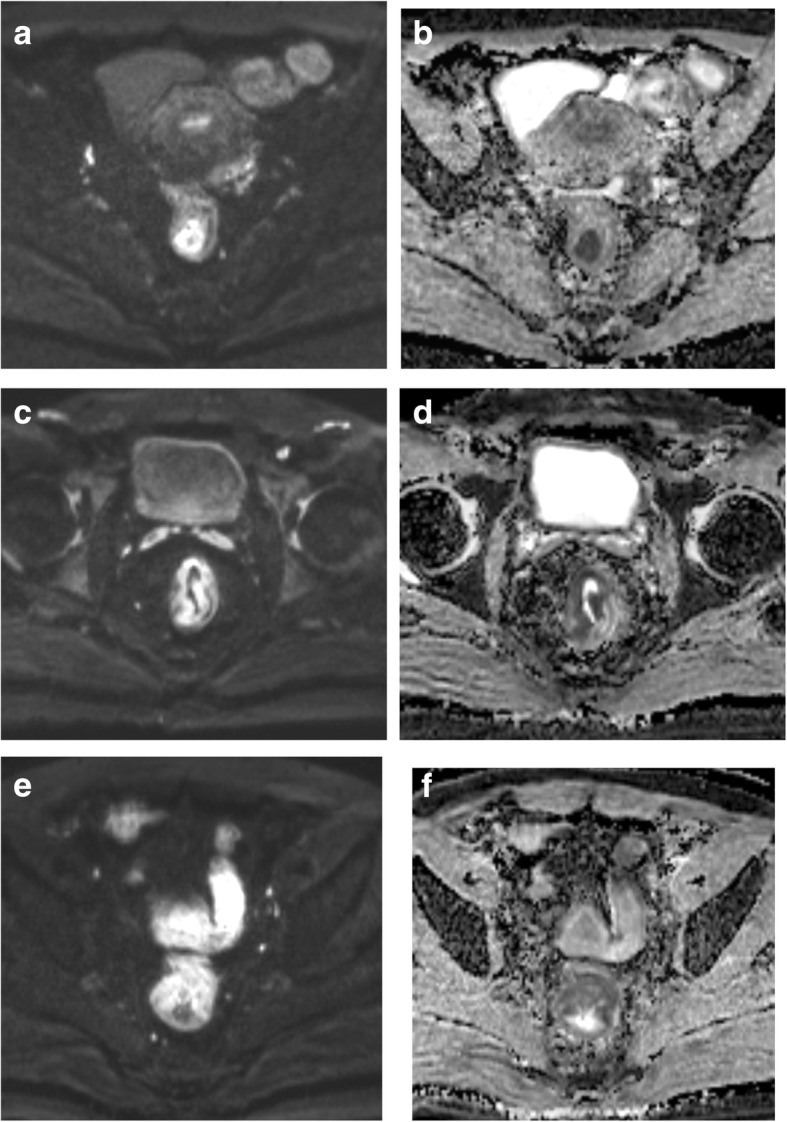
**a**, **b** A 53-year-old woman with moderately differentiated rectal adenocarcinoma that was eminence type. **c**, **d** A 68-year-old man with moderately differentiated rectal adenocarcinoma that was ulcer type. **e**, **f** A 64-year-old man with mucinous adenocarcinoma. **a**, **c**, **e** Axial RESOLVE images in tumour section (*b* = 800 s/mm^2^). **b**, **d**, **f** Measurement of the ADC values for each tumour on the ADC maps. **d**, **b** The ADC values in tumours with ulcer type (0.82 × 10^−3^ mm^2^/s) were higher than that in eminence type (0.68 × 10^−3^ mm^2^/s). **f**, **b**, **d** The ADC values in mucinous adenocarcinoma (1.04 × 10^−3^ mm^2^/s) were higher than that in ductal adenocarcinoma

**Fig. 3:**
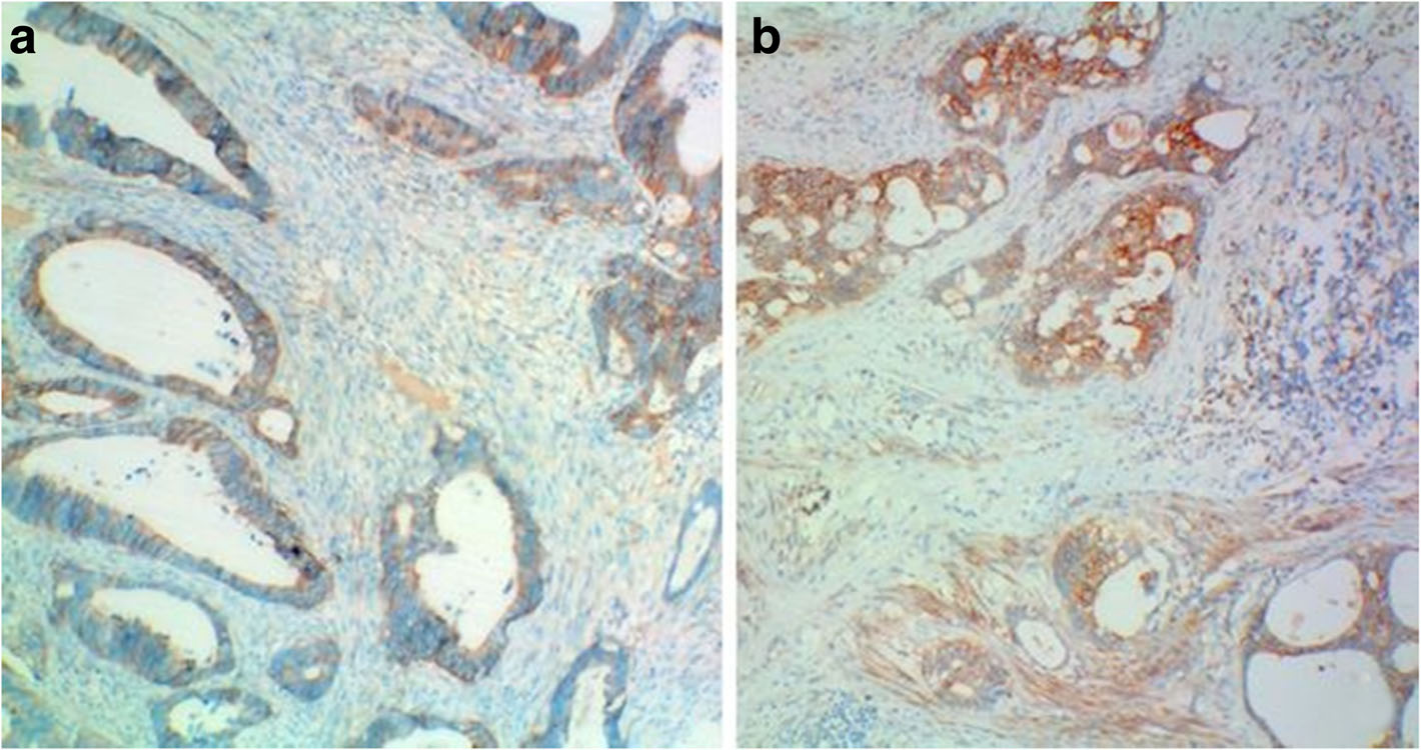
(× 100) Histopathologic specimen of rectal adenocarcinoma showed negative EGFR expression (**a**) and positive EGFR expression (**b**). The RESOLVE ADC value of rectal adenocarcinoma with negative EGFR expression (**a**) was 0.807 × 10^−3^ mm^2^/s, higher than the ADC values (0.732 × 10^−3^ mm^2^/s) of positive EGFR expression (**b**)

**Fig. 4:**
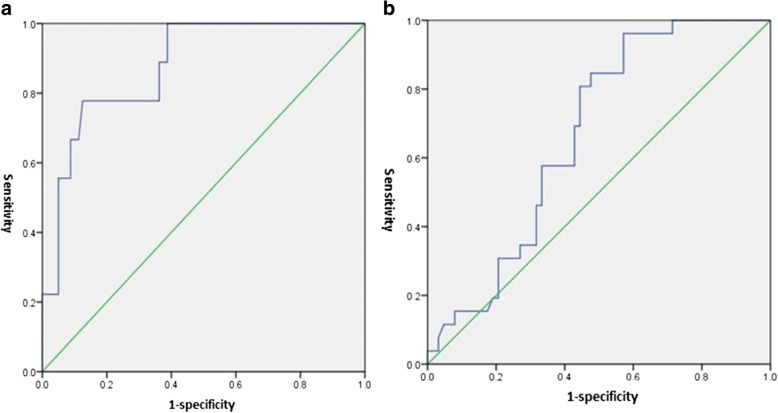
**a** Receiver operator characteristic (ROC) curve of mucinous adenocarcinoma in RAC. **b** ROC curve of EGFR negative expression in RAC

In the 89 RAC cases, the RESOLVE ADC values in the ulcer-type group were significantly higher than those in the eminence-type group (*P* = 0.001), and there were statistically significant differences in the RESOLVE ADC values between the different pathological types of RAC (*P* = 0.037) (Fig. 2). The RESOLVE ADC values in the EGFR-positive group were significantly lower than those in the EGFR-negative group (*P* = 0.028) (Fig. 3). No significant differences in the RESOLVE ADC values were identified between the different degrees of pathologic differentiation, TN stages, and positive or negative lymph nodes (*P* > 0.05) (Table 2). ROC (a) showed that using a tumour RESOLVE ADC value > 0.922 × 10^−3^ mm^2^/s as the threshold value, the diagnostic sensitivity and specificity were 80 and 90% for mucinous adenocarcinoma, respectively, and the AUC was 0.877; ROC (b) showed that using a tumour RESOLVE ADC > 0.764 × 10^−3^ mm^2^/s as the threshold, the diagnostic sensitivity and specificity for EGFR-negative in RAC was 60 and 70%, respectively, and the AUC was 0.665 (Fig. 4).

**Table 2:** The differences in pretreatment tumour RESOLVE ADC values stratified according to different histopathological prognostic characteristics

Prognostic factors	Groups	Patients (*n*)	RESOLVE ADC (± SD) (10^−3^ mm^2^/s)	*P* value
Gross classification	Ulcer	56	0.810 ± 0.126	0.001
	Eminence	33	0.715 ± 0.111	
Histological type	Tubular	64	0.744 ± 0.104	0.000
	Papillary	16	0.795 ± 0.120	
	Mucinous	9	0.958 ± 0.155	
Pathologic differentiation	High	16	0.792 ± 0.161	0.844
	Moderate	63	0.771 ± 0.123	
	Poor	10	0.769 ± 0.129	
T stage	T1	5	0.804 ± 0.134	0.876
	T2	36	0.775 ± 0.136	
	T3–4	48	0.772 ± 0.124	
N stage	N0	56	0.768 ± 0.136	0.526
	N+	33	0.786 ± 0.117	
Cancer nodule	Positive	10	0.746 ± 0.106	0.446
	Negative	79	0.778 ± 0.132	
EGFR	Positive	63	0.756 ± 0.131	0.028
	Negative	26	0.822 ± 0.113

## Discussion

### Comparison of RESOLVE ADC values between different pathological prognostic factors in RAC

There are three pathological types of RAC: ductal, mucinous, and papillary. The guidelines established by the National Comprehensive Cancer Network (NCCN) consider that the therapeutic decision of mucinous tumours is similar to non-mucinous tumours and the histology does not affect the treatment strategies. However, due to the unique biological behaviour of rectal mucinous adenocarcinoma, the clinical treatment of mucinous adenocarcinoma should differ from tubular adenocarcinoma and papillary adenocarcinoma. Some researchers [16] also believe that the surgical strategy for mucinous adenocarcinomas should differ from ductal and papillary adenocarcinomas, and therefore, mucinous adenocarcinoma should be considered as an independent prognostic factor. Surgical treatment of colorectal mucinous adenocarcinoma should emphasise the principle of extended radical mastectomy, and radiotherapy should be carefully chosen [17]. The histopathology of mucinous adenocarcinoma shows a large amount of mucus in the epithelial cancer cells. It is believed that mucinous tumours are associated with young adult patients. The growth rate of the tumour is faster, so the tumour volume is usually large. The tumour tends to infiltrate the surrounding tissues, and lymph node metastasis is more common [18, 19]. In addition, it is less sensitive to radiochemotherapy, and the prognosis is usually poor [19]. Therefore, whether preoperative rectal cancer belongs to mucinous adenocarcinoma is beneficial to developing a more scientific treatment plan, assessing the prognosis of patients, adjusting treatment in time, and improving the prognosis of patients. The specimens obtained from preoperative enteroscopy are usually too small to provide an accurate histological type. The existence of a lesion limits the diffusion of water molecules and therefore presents as a high signal on DWI. In rectal cancer, the nuclei of the tumour cells are enlarged, the nucleoplasm is reduced, and the nucleus/nucleoplasm ratio is increased, which decreases the extra- and intra-cellular spaces compared to normal cells, thus limiting the diffusion of water molecules in the tumour tissues and decreasing the ADC values compared to normal tissues. The present study found that although mucinous adenocarcinoma presented diverse RESOLVE and ADC images, its RESOLVE ADC values were significantly higher than ductal and papillary adenocarcinomas, which is consistent with most previous studies. Some researchers [9] reported that the histological type of rectal cancer could affect the ADC values because the density of mucin tumour cells is low and the extracellular content of mucin is high. Katsuhiro et al. [20] noted that the greater proportion of mucinous cancer is mucus rather than cellular composition because it is composed of cancer cells that secrete mucus, and therefore, it presents an increased signal of mucus on MRI. The mean ADC value of mucinous cancer in Katsuhiro’s study was 1.49 ± 0.34 × 10^−3^ mm^2^/s vs 0.958 ± 0.155 × 10^−3^ mm^2^/s in our study. The possible reason for the great discrepancy between the two studies may be due to the different methods of ADC measurement. In this study, ADC values were measured using RESOLVE and contrast-enhanced images in combination and restricted ROI to the central area of the tumour lesion. Less image distortion is observed by applying RESOLVE method during diffusion imaging because this technique divides the *k*-space into multiple segments to shorten the echo spacing [12, 14, 15]. In order to remove the phase artefacts caused by motion, a navigator echo is also applied during data acquisition [21]. This method is simple, and the ADC results are more reliable. This study found that the RESOLVE ADC values in ductal carcinoma were lower than in papillary carcinoma, but the difference was not statistically significant. We therefore conclude that the RESOLVE ADC value greater than 0.866 × 10^−3^ mm^2^/s as shown by ROC analysis could be used to differentiate between mucinous and non-mucinous adenocarcinoma in the context of the imaging presentation, thus providing clues for selecting the therapeutic approach and predicting the invasiveness and prognosis of RAC.

Gross classification of RAC includes the eminence type, ulcer type (limited ulcer type and infiltration ulcer type), and diffuse type. Eminence-type RAC grows in a collisional, outward, and propulsion-to-lumen manner, slowly infiltrating the intestinal wall, and therefore, LN metastasis is less likely to occur in this type. This type of RAC mostly presents symptoms of obstruction, and the prognosis is relatively good. In ulcer-type RAC, the tumour infiltrates the intestinal wall while it is proliferating, and therefore, LN metastasis occurs early in this type of RAC, which causes stenosis of the intestinal cavity, early metastasis, and poor prognosis. The diffuse type of RAC is relatively rare. However, cases with early metastasis, poor prognosis, and death are not rare in eminence-type RAC [22]. Therefore, it is difficult to use gross classification alone to predict the prognosis of patients with RAC. Comparison of different gross types with ADC values in our study showed that RESOLVE ADC values in eminence-type RAC were significantly lower than in ulcer-type RAC. Therefore, it is worth further investigating the significance of a combination of gross classifications with RESOLVE ADC values in judging the clinical symptoms, pathological changes, and prognosis in RAC patients.

The results of studies about the correlation between ADC values and the degree of tumour differentiation and TN stage in RAC are controversial. More researchers [9–12] believed that the ADC values were different in RAC of different degrees of differentiation; the ADC values in low differentiation tumours were relatively low, which is consistent with our findings in the present study. However, the difference was not statistically significant. The possible reason is that the number of cases with high and poor differentiation cases was relatively small in our study. In addition, to avoid the inaccuracy of preoperative staging, all of the T and N stages discussed in this study were based on postoperative pathology. The result showed that there was no significant difference in RESOLVE ADC values between different T stages, nor was there a significant difference in RESOLVE ADC values between the presence and absence of LN metastasis. These findings are consistent with the results of Akashi et al. [9]. Other studies reported that there were some differences in ADC values between different mrN stages in RAC patients. Curvo-Semedo et al. [10] reported that ADC values in mrN^+^ tumours were even lower than in mrN0 tumours, but no statistically significant difference in ADC values was found between different mrT stages. The reason for these varying results may be due to the differences between preoperative and postoperative TN stages or due to the small number of cases. In addition, measurement of the ADC value may also be affected by the person who performed the ADC measurement, because there is no standardised method for ADC measurement at present, and therefore, further study in this field is required.

### ADC differences between different levels of EGFR expression in RAC

EGFR, which belongs to the tyrosine kinase type I receptor family, is extensively expressed in the epidermal cells, stromal cells, partial glial cells, and smooth muscle cells, playing an important role in regulating cell growth and tissue repair. The cellular effects of the EGFR signalling system include cell proliferation, migration, and adhesion. EGFR over-expression was observed in multiple malignant tumours originating from epithelial cells. Abnormal activation or over-expression of EGFR often induces malignant cell transformation and therefore is closely associated with the development, progression, malignancy, and prognosis of RAC [23]. Some studies [24] have revealed that EGFR-positive rectal cancer cells are more proliferative and infiltrative, which correlate with the TNM stage and LB metastasis, indicating that the high expression of EGFR is associated with the high invasiveness and metastasis of tumours. According to the statistical data reported in the current literature, there are relatively large differences in the expression rates of EGFR in RAC, reporting that the over-expression of EGFR was observed in 65–97% patients with RAC [25, 26]. In the present study, we found an 80% positive expression rate of EGFR in the patients with RAC. We also found that RESOLVE ADC values in patients with positive EGFR expression were significantly lower than in patients with negative EGFR expression, suggesting that RESOLVE ADC values could be used as independent factors for predicting positive EGFR expression and markers for judging disease progression and prognosis. EGFR has become an important therapeutic target of RAC. The clinical application of cetuximab and panitumumab has further confirmed the important role of EGFR in the development and progression of RAC [27, 28]. It is therefore expected that the measurement of ADC values through preoperative RESOLVE-MR could be used to predict the response sensitivity of RAC to anti-EGFR target drugs.

There are several limitations to this study. It was a retrospective study that may be prone to selection bias. The small number of patients with each tumour stage and the exclusion of patients with distant metastases limited the applicability of the results. Thus, large prospective multi-centre trials are necessary to fully evaluate the function of RESOLVE-DWI on the biological behaviour of rectal adenocarcinoma.

## Conclusion

RESOLVE can obtain high SNR images and reduce the distortion caused by a magnetic susceptibility effect, which greatly reduces the deformation of the DWI images and improves the stability of the ADC values. RESOLVE ADC values are correlated with pathological prognostic factors in RAC, especially with respect to EGFR expression. The quantitative description of ADC values of RESOLVE could be used to assess the biological behaviour of rectal adenocarcinoma and serve as molecular markers.

All procedures performed in studies involving human participants were in accordance with the ethical standards of the institutional and/or national research committee and with the 1964 Helsinki Declaration and its later amendments or comparable ethical standards.

## Abbreviations

ADC: Apparent diffusion coefficient
AJCC7: American Joint Committee on Cancer
CRC: Colorectal cancer
DWI: Diffusion-weighted imaging
DWI-MR: Diffusion-weighted magnetic resonance imaging
LN: Lymph node
RAC: Rectal adenocarcinoma
RESOLVE: Readout-segmented echo-planar diffusion-weighted imaging
ROI: Region of interest
SD: Standard deviation
